# Relationship between dietary consumption of live microbes with mortality in adults with chronic kidney disease

**DOI:** 10.1007/s40620-025-02212-w

**Published:** 2025-02-12

**Authors:** Jianxin Han, Huan Zhang, Xinchun Li, Yumei Tang, Yunfei Du, Haiyan Zhang, Dan Liao

**Affiliations:** 1 Department of Nephrology, The People’s Hospital of Renshou County, The Second People’s Hospital of Meishan City, Meishan, China; 2 North Sichuan Medical College, Nanchong, China; 3 Chengdu Medical College, Chengdu, China; 4 Pingshan County Hospital of Chinese Medicine, Yibin, Sichuan China; 5 Department of Nephrology, Mianyang Central Hospital, School of Medicine, University of Electronic Science and Technology of China, Mianyang, China

**Keywords:** Chronic kidney disease, Dietary live microbes, Gut dysbiosis, Mortality

## Abstract

**Background:**

The connection between gut dysbiosis and chronic kidney disease (CKD) has been recognized, but, the effect of dietary intake of live microbes on the prognosis of CKD is still unclear. This analysis examined the relationship of dietary live microbe intake with mortality among adults with CKD.

**Methods:**

For this study, information was gathered from the National Health and Nutrition Examination Survey 1999–2018, which included 8725 adult participants with CKD. MedHi refers to the live microbial content of food beyond 10^4^ CFU/g. To elucidate the link between MedHi dietary live microbe intake and mortality from all-cause and cardiovascular disease (CVD), we implemented a weighted multivariate Cox regression analysis.

**Results:**

In contrast to survivors, non-survivors had a lower intake of dietary live microbes. The findings from the multivariable model indicated a negative and linear relationship between an increment of 100 g in MedHi foods and reduced mortality from all-causes and CVD. Likewise, participants in the highest MedHi food group exhibited a 20% and 26% decreased risk of all-cause and CVD mortality, respectively, compared to those in the lowest MedHi food group. Stratified analyses conducted on various subgroups yielded consistent findings.

**Conclusion:**

A significant inverse linear relationship was found between high dietary live microbe consumption and reduced all-cause and CVD mortality.

**Graphical abstract:**

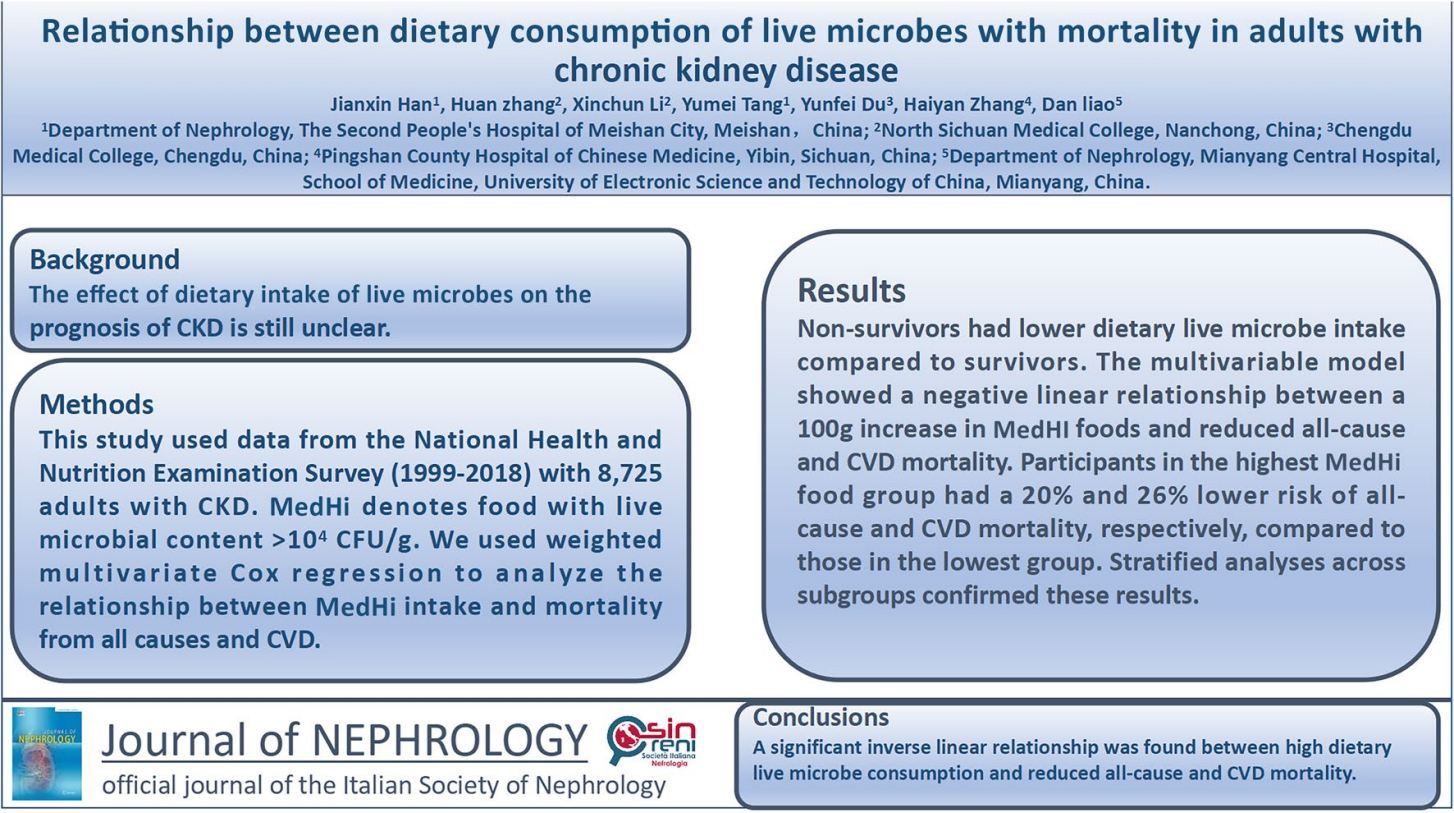

**Supplementary Information:**

The online version contains supplementary material available at 10.1007/s40620-025-02212-w.

## Introduction

Chronic kidney disease (CKD) is a significant global health challenge, with an estimated global prevalence of about 10% and over 800 million affected individuals [1]. Notably, the prevalence of CKD is higher in the United States (US), reaching approximately 15%, primarily attributed to the increased prevalence of hypertension, diabetes, and cardiovascular disease (CVD) [2]. In 2017 alone, CKD accounted for 1.2 million deaths, and between 1990 and 2017, the global mortality rate associated with CKD increased by 41.5% [3]. Therefore, identifying modifiable factors is crucial for preventing CKD or delaying premature death.

CKD is characterized by dysbiosis and accumulation of toxic metabolites [4, 5]. Specifically, the increase in intestinal urea concentration can result in alterations in gut microbiota, and in turn, an increase in pathogenic gut microbiota can lead to an increase in uremic toxins [6]. There is growing recognition of the potential of dietary interventions targeting the gut microbiota-gut-kidney axis to delay the progression of CKD [7]. A recent cross-sectional survey has indicated a potential association between the consumption of probiotics, prebiotic supplements, or yogurt and a reduced risk of CKD and its progression [8]. A recent meta-analysis has suggested that non-dietary probiotics, prebiotics, and synbiotics have the potential to reduce inflammation, oxidative stress, and blood lipids in CKD patients [9]. Live microbes exist in various foods, including unpeeled fruits and vegetables, as well as fermented dairy products [10]. These foods are easy to obtain, inexpensive, and can be consumed regularly. Previous studies have shown that dietary live microbes can survive in the digestive tract contributing to the improvement of gut microbiota and modulation of the immune system [11, 12].

However, the existing literature does not provide sufficient evidence regarding whether dietary live microbes can improve the prognosis of CKD patients. Thus, the primary aim of this study was to elucidate the relationship between dietary live microbe consumption and the occurrence of all-cause mortality and CVD mortality in adults diagnosed with CKD in the US. This will contribute to the development of novel interventions aimed at improving the prognosis of CKD patients.

## Methods

### Study population

The NHANES study is carried out every two years by the National Center for Health Statistics (NCHS) to estimate the nutritional and health conditions of the entire population of the US employing intricate and multi-level probability sampling design methods.

CKD is characterized by an estimated glomerular filtration rate (eGFR) < 60 mL/min/1.73 m^2^ and/or urinary albumin to creatinine ratio (UACR) ≥ 30 mg/g, as per the KDIGO 2021 guideline [13]. The eGFR was computed through the CKD-Epidemiology Collaboration equation, which includes serum creatinine, age, sex, and race/ethnicity [14]. The calculation for eGFR was as follows: 141 × min (SCr/κ, 1)^α^ × max (SCr/κ, 1)^−1.209^ × 0.993^Age^ × 1.018 if female × 1.159 if Black, where SCr represents serum creatinine, κ is 0.7 for females and 0.9 for males, α is − 0.329 for females and − 0.411 for males, min denotes the lesser value between SCr/κ or 1, and max signifies the greater value between SCr/κ or 1. In the present analysis, we initially identified CKD participants aged 20 and above from a series of 10 consecutive cycles spanning from 1999 to 2018. Afterward, we removed pregnant females and individuals who did not have 24-h dietary recalls and follow-up information, which led to a total of 8725 participants in the final sample size (Supplementary Fig. 1).

### Covariates

Given possible variables that could affect the results, this study incorporated several covariates to clarify any uncertainty in the correlation between the consumption of dietary live microbes and mortality. Demographic parameters, such as age, gender, race/ethnicity (including Black, White, Mexican, and others), body mass index (BMI), educational attainment (ranging from below high school to above high school), and poverty income ratio (classified as < 1.3, 1.3–3.5, and ≥ 3.5), were collected through questionnaires. BMI was determined through anthropometric measurements. The participants self-reported their habits and health conditions, encompassing smoking habits (never, former, current), alcohol intake (none, moderate, heavy, and binge), levels of physical activity (none, moderate, and vigorous), hypertension, diabetes, hyperlipidemia, and CVD. The diagnosis of diabetes met one of four different criteria: self-reported diagnosis, the use of diabetes medications, a hemoglobin A1c ≥ 6.5%, or a fasting plasma glucose ≥ 126 mg/dL. The definition of hyperlipidemia was based on three distinct criteria: (1) the utilization of lipid-lowering drugs; (2) triglyceride levels equal to or greater than 150 mg/dL; (3) total cholesterol levels equal to or greater than 200 mg/dL, with high-density lipoprotein cholesterol below 40 mg/dL for males or below 50 mg/dl for females, or low-density lipoprotein cholesterol levels equal to or greater than 130 mg/dL. Hypertension was determined through the use of antihypertensive drugs or systolic/diastolic blood pressure ≥ 140/90 mmHg. CVD was ascertained by self-reported presence of one of five subtypes from among congestive heart failure, coronary heart disease, angina, heart attack, or stroke. Additional covariate indicators included Healthy Eating Index-2015 (HEI-2015) and laboratory data, including UACR and serum creatinine. The HEI-2015 score ranges between 0 and 100 points. A higher score reflects healthier eating.

### Mortality

The Public Use Linked Mortality File was used to determine the outcomes of the participants including data on their survival status from the National Death Index until December 31st, 2019. The primary cause of death was identified following ICD-10 codes, encompassing deaths caused by CVD (I00-I09, I11, I13, I20-I51, and I60-I69) and other causes.

### Estimating dietary live microbe intake

Dietary data are collected using an in-person 24-h dietary recall. NHANES dietary data were obtained from the What We Eat in America (WWEIA) program conducted by the United States Department of Agriculture (USDA), which aims to provide the nutrient values for foods and beverages reported in WWEIA (https://www.ars.usda.gov/northeast-area/beltsville-md-bhnrc/beltsville-human-nutrition-research-center/food-surveys-research-group/). The method for determining dietary live microbes was performed as before [10], and has been employed in multiple studies [15, 16]. In short, to estimate the number of live microbes per gram of food corresponding to the 9388 food codes from 48 subgroups in the NHANES database, four experts (Maria L. Marco, Mary E. Sanders, Robert Hutkins, and Colin Hill) conducted evaluations of each food item. Their assessments were based on a comprehensive review of existing literature, authoritative reviews, and the known impacts of food processing techniques, such as pasteurization, on microbial viability. The differences in each food classification were established through internal team consultations and validated by external consultation with Fred Breidt, a microbiologist at the USDA Agricultural Research Service. Based on the expected number of live microbes, the food was classified as low (< 10^4^ CFU/g), medium (10^4^–10^7^ CFU/g), or high (> 10^7^ CFU/g). Foods with low microbial content primarily consisted of pasteurized products, foods with moderate microbial content mainly included unpeeled fresh fruits and vegetables, and foods with high microbial content included unpasteurized fermented foods. MedHi refers to food classified as having moderate or high microbial content, and we calculated the grams of food classified as MedHi for each individual. The classification of each food item was documented in the prior study [10].

### Statistical analysis

Following the NHANES analysis guidelines, we employed sampling weights (WTDRD1) and masked variance in R 4.2.2 to account for the intricate study design of NHANES. We examined the characteristics of individuals with and without all-cause mortality using Student’s *t*-tests for continuous factors and chi-square tests for categorical factors. In regression analysis, our primary focus was to investigate the correlation between exposure to MedHi dietary intake and the rate of mortality. Thus, we conducted an examination of MedHi as both a continuous and categorical variable. The categorization involved dividing the participants into three groups based on tertile distribution: Tertile 1 referred to individuals who did not consume any live microbe foods classified as Med and Hi, the Tertile 2 group included individuals whose consumption of MedHi foods was in the range of 0–110 g/d, and the Tertile 3 group consisted of individuals with consumption exceeding 110 g/d. Weighted multivariate Cox regression analysis was adopted to examine the relationship between MedHi dietary live microbe consumption and mortality across three different models. In Model I, adjustments were made for age, gender, and race/ethnicity. Model II incorporated age, gender, race/ethnicity, educational attainment, poverty income ratio, BMI, smoking habits, alcohol consumption, and physical activity levels, while Model III further incorporated health conditions (hypertension, diabetes, hyperlipidemia, and CVD), serum creatinine, UACR and HEI-2015. Restricted cubic splines (RCS) with four knots based on multivariate regression analysis were implemented to visually represent the linear or nonlinear relationship between MedHi dietary active microbe intake and mortality rate. Stratified analyses were implemented to verify the robustness of our findings based on gender, age, hyperlipidemia, hypertension, diabetes, and CVD. Additionally, we implemented several sensitivity analyses. Firstly, since prebiotic/probiotic supplements were not included in the dietary live microbes, we further adjusted for Prebiotic/Probiotic supplements (only information concerning 4659 participants was available). Secondly, given that unpasteurized foods contain a large amount of vitamins, we further adjusted for dietary factors including vitamin A, vitamin C, vitamin E, and carotenoids. Thirdly, to reduce the risk of reverse causality bias, the analysis excluded individuals who died within a 2-year time frame. Fourthly, survival times were censored at the latest at 15 years to ensure consistency. Finally, individuals with implausible energy intake levels falling below 500 kcal or exceeding 4000 kcal per day were excluded from the analysis. A *p* < 0.05 showed statistical significance.

## Results

A cohort of 8725 individuals aged over 20 years were diagnosed with CKD. During a median follow-up duration of 84 months, a total of 3501 deaths from various causes and 1262 deaths specifically due to CVD were recorded. Table 1 presents an overview of the main characteristics of the included population, categorized by presence or absence of all-cause mortality. Compared to individuals who survived, those who did not were found to have a higher likelihood of consuming lower amounts of dietary live microbes, regardless of whether the levels of live microbes were low, medium, high, or MedHi (all *p* < 0.05). Non-survivors, in comparison to survivors, tended to be older, male, White, separated, former smokers, moderate drinkers, physically inactive, with lower levels of educational achievement, family income, and HEI-2015 score. Additionally, they showed a higher occurrence of hypertension, diabetes, hyperlipidemia, and CVD (all *p* < 0.001).

**Table 1:** Participant characteristics in NHANES 1999–2018, weighted

Variable	Total	Survivors	Non-survivors	*P*
	*N* = 8725	*N* = 5224	*N* = 3501	
Age (years)	60.59 (0.47)	53.32 (0.59)	71.84 (0.46)	< 0.001
Sex (%)				< 0.001
Male	43.38 (0.01)	41.39 (1.04)	47.30 (0.99)	
Female	56.62 (0.02)	58.61 (1.04)	52.70 (0.99)	
Race/ethnicity (%)				< 0.001
Black	12.46 (0.01)	13.10 (0.86)	11.19 (0.86)	
White	70.11 (0.03)	66.03 (1.39)	78.17 (1.35)	
Mexican	6.61 (0.01)	8.08 (0.66)	3.71 (0.57)	
Other	10.83 (0.01)	12.80 (0.80)	6.93 (0.86)	
BMI (kg/m^2^)	29.29 (0.18)	29.50 (0.22)	28.96 (0.26)	0.082
Education level (%)				< 0.001
Less than high school	24.96 (0.01)	20.18 (0.78)	34.50 (1.29)	
High school graduates	26.17 (0.01)	26.00 (0.93)	26.61 (0.96)	
Above high school	48.75 (0.02)	53.82 (1.13)	38.89 (1.21)	
Marital status (%)				< 0.001
Separated	42.92 (0.01)	39.46 (1.06)	51.06 (1.18)	
Married	56.16 (0.02)	60.54 (1.06)	48.94 (1.18)	
PIR (%)				< 0.001
< 1.3	24.45 (0.01)	25.07 (0.91)	29.39 (1.34)	
1.3–3.5	38.13 (0.01)	38.60 (0.99)	46.83 (1.22)	
≥ 3.5	29.61 (0.01)	36.33 (1.23)	23.79 (1.24)	
Smoking status (%)				< 0.001
Never	49.73 (0.01)	53.39 (1.01)	42.61 (1.16)	
Former	32.69 (0.01)	28.53 (0.83)	40.99 (1.20)	
Current	17.50 (0.01)	18.08 (0.84)	16.40 (0.88)	
Alcohol consumption (%)				< 0.001
None	21.52 (0.01)	20.34 (0.89)	26.79 (1.24)	
Moderate	46.54 (0.01)	46.47 (1.23)	52.99 (1.21)	
Heavy	15.20 (0.01)	19.32 (0.79)	9.09 (0.75)	
Binge	12.39 (0.01)	13.87 (0.83)	11.14 (0.62)	
Physical activity (%)				< 0.001
Never	58.42 (0.02)	54.07 (0.99)	67.05 (1.19)	
Moderate	25.11 (0.01)	25.34 (0.83)	24.66 (1.02)	
Vigorous	16.45 (0.01)	20.58 (0.81)	8.29 (0.67)	
Hypertension (%)	68.92 (0.02)	61.11 (1.06)	84.35 (0.78)	< 0.001
Diabetes (%)	33.15 (0.01)	29.72 (0.93)	39.92 (1.02)	< 0.001
Hyperlipidemia (%)	80.91 (0.02)	79.49 (0.86)	83.73 (0.73)	< 0.001
CVD (%)	24.97 (0.01)	16.36 (0.65)	42.02 (1.19)	< 0.001
UACR (mg/g)	43.16 (1.03)	41.31 (1.03)	47.23 (1.04)	< 0.001
Serum creatinine (mg/dl)	1.02 (1.01)	0.96 (1.01)	1.16 (1.01)	< 0.001
HEI-2015	51.57 (13.58)	52.24 (13.05)	51.22 (13.82)	0.016
Low (grams/d)	2667.28 (49.78)	2906.71 (71.79)	2296.70 (52.97)	< 0.001
Med (grams/d)	97.50 (3.72)	104.40 (5.26)	86.82 (4.30)	0.009
Hi (grams/d)	15.87 (1.34)	17.36 (1.66)	13.57 (2.13)	0.046
MedHi (grams/d)	113.37 (4.44)	121.76 (6.04)	100.39 (5.34)	0.005

BMI, body mass index; PIR, poverty-to-income ratio; CVD, cardiovascular disease; UACR, urinary albumin to creatinine ratio; HEI-2015, healthy eating index-2015; Low: < 10^4^ CFU/g foods; Med: 10^4^–10^7^ CFU/g foods; Hi: > 10^7^ CFU/g foods; MedHi: > 10^4^ CFU/g foods

**Fig. 1:**
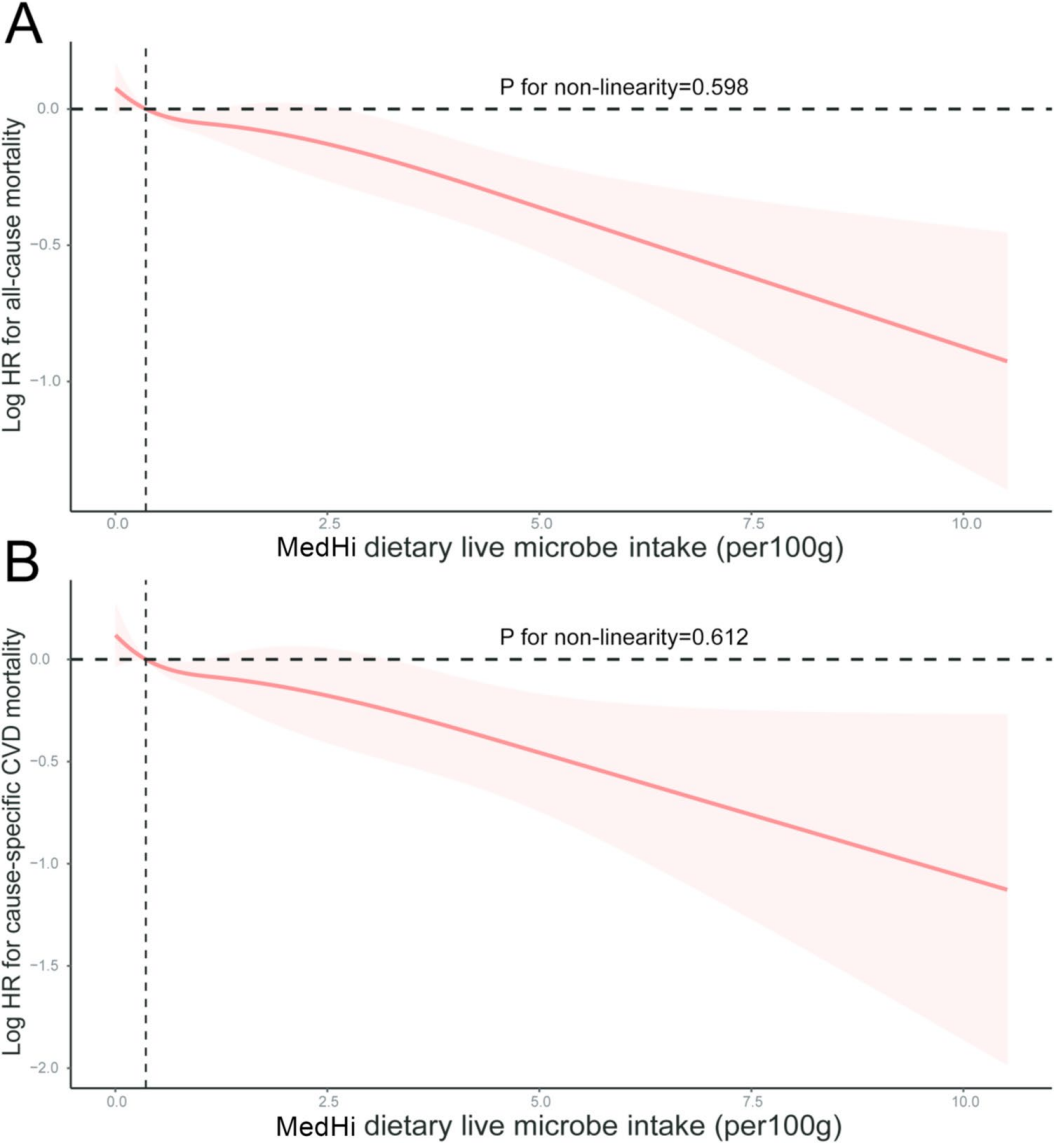
Restricted cubic spline presented the linear association between the consumption of MedHi foods and mortality. A clear dose–response relationship between the consumption of MedHi foods and all causes (**A**) and CVD (**B**) mortality were obtained. HR, hazard ratios; MedHi: > 10^4^ CFU/g foods; CVD, cardiovascular diseases

The association between MedHi dietary live microbe intake and mortality was examined using weighted multivariate Cox regression analyses, as shown in Table 2. According to the multivariable model, the highest MedHi food group (Tertile 3) exhibited a 20% reduction in all-cause mortality risk and a 26% reduction in CVD mortality risk compared to the lowest MedHi food group (Tertile 1) (p for trend < 0.05). The results depicted in Fig. 1 demonstrate a clear dose–response relationship between the consumption of MedHi foods and all causes and CVD mortality (*p* for non-linearity > 0.05). After considering all pertinent factors, each 100 g increment in MedHi foods was negatively linked to a reduced risk of all-cause and CVD mortality.

**Table 2:** Association of MedHi dietary live microbe intake (per 100 g) with the risk of all-cause and cardiovascular mortality

	MedHi			P for trend	Per one-unit increment in MedHi
	Tertile 1	Tertile 2	Tertile 3		
	HR (95% CI)	HR (95% CI)	HR (95% CI)		
All-cause mortality					
Model I	Reference	0.80 (0.70, 0.91)^#^	0.72 (0.65, 0.81)^#^	< 0.001	0.91 (0.88, 0.94)^#^
Model II	Reference	0.90 (0.78, 1.02)	0.83 (0.74, 0.94)^†^	0.002	0.93 (0.89, 0.97)^#^
Model III	Reference	0.90 (0.78, 1.03)	0.80 (0.70, 0.91)^†^	0.002	0.92 (0.88, 0.96)^†^
CVD mortality					
Model I	Reference	0.80 (0.65, 0.98)^*^	0.68 (0.57, 0.81)^#^	< 0.001	0.88 (0.83, 0.93)^#^
Model II	Reference	0.91 (0.72, 1.14)	0.79 (0.65, 0.96)^*^	0.020	0.91 (0.85, 0.97)^†^
Model III	Reference	0.90 (0.70, 1.16)	0.74 (0.59, 0.93)^*^	0.020	0.89 (0.83, 0.96)^†^

MedHi: > 10^4^ CFU/g foods; HR, hazard ratios; 95% CI, 95% confidence interval

Model I adjusted for age, sex, and race/ethnicity

Model II adjusted for model I + education level, marital status, PIR, smoking status, alcohol, physical activity, and body mass index

Model III adjusted for model II + hypertension, diabetes, hyperlipidemia, CVD, serum creatinine, UACR and HEI-2015

Tertile 1 refers to individuals who consumed no live microbe food classified as MedHi. Tertile 2 includes individuals who consumed MedHi in the 0–110 g/d. Tertile 3 consists of individuals with consumption exceeding 110 g/d

*p < 0.05, ^†^p < 0.01, ^#^p < 0.001

To evaluate whether various subgroups modified the association between MedHi dietary live microbe intake and mortality, we performed stratified analyses (Table 3). The subgroups, categorized by age (< 65 or ≥ 65), sex (female or male), and the presence or absence of hypertension, diabetes, and hyperlipidemia, did not exhibit any significant effect on the association between MedHi dietary live microbe consumption and all-cause mortality (all *P*_interaction_ > 0.05). Interestingly, this association appeared to be more pronounced in patients with CVD compared to those without CVD (*P*_interaction_ = 0.023).

**Table 3:** Subgroup analysis for the association between MedHi dietary live microbe intake (per 100 g) and all-cause mortality

	MedHi			*P* for trend	*P* for interaction
	Tertile 1	Tertile 2	Tertile 3		
	HR (95% CI)	HR (95% CI)	HR (95% CI)		
Age					0.306
< 65 years	Reference	0.81 (0.54, 1.22)	0.95 (0.60, 1.59)	0.754	
≥ 65 years	Reference	0.92 (0.79, 1.06)	0.77 (0.67, 0.89)^#^	< 0.001	
Sex					0.364
Male	Reference	0.88 (0.73, 1.06)	0.73 (0.61, 0.88)^†^	0.002	
Female	Reference	0.94 (0.79, 1.13)	0.89 (0.73, 1.09)	0.259	
Hypertension					0.496
Yes	Reference	0.87 (0.74, 1.01)	0.78 (0.68, 0.89)^†^	< 0.001	
No	Reference	1.03 (0.77, 1.38)	0.91 (0.66, 1.26)	0.582	
Diabetes					0.109
Yes	Reference	0.94 (0.77, 1.16)	0.93 (0.77, 1.10)	0.386	
No	Reference	0.87 (0.74, 1.02)	0.72 (0.60, 0.86)^#^	< 0.001	
Hyperlipidemia					0.223
Yes	Reference	0.88 (0.76, 1.03)	0.84 (0.73, 0.96)^†^	0.008	
No	Reference	0.98 (0.72, 1.35)	0.65 (0.46, 0.96)^*^	0.020	
CVD					0.023
Yes	Reference	0.72 (0.59, 0.87)^#^	0.79 (0.66, 0.94)^†^	0.005	
No	Reference	1.02 (0.87, 1.20)	0.86 (0.73, 1.02)	0.080	

MedHi: > 10^4^ CFU/g foods; HR, hazard ratios; 95% CI, 95% confidence interval; CVD, cardiovascular disease

Tertile 1–3 group as in Table 2

*p < 0.05, ^†^p < 0.01, ^#^p < 0.001

In the conducted sensitivity analyses in Supplementary Table 1, the inclusion of prebiotic/probiotic supplements, or dietary factors (vitamin A, vitamin C, vitamin E, and carotenoids) in the adjusted full model did not alter the negative correlation between MedHi foods and both all-cause and CVD mortality. Consistent results were obtained when we implemented a censoring mechanism at the 15-year follow-up. When individuals with extreme energy intake were excluded, the results remained consistent. Upon excluding adult CKD individuals who died within the initial two years, the correlation between MedHi food and all-cause mortality did not undergo any material alteration. However, the correlation between MedHi food and CVD mortality did not reach statistical significance.

## Discussion

Our findings show an inverse linear relationship between high intake of dietary live microbes and risk of mortality from any cause and for CVD. Furthermore, stratified analyses and multiple sensitivity analyses confirmed this relationship.

The primary focus of previous studies has mainly been on probiotics and dairy products that have undergone fermentation as sources of live microorganisms, suggesting a positive impact on kidney disease. A cross-sectional study revealed a significant relationship between regular consumption of yogurt and/or probiotics and reduced odds of proteinuria [17]. Furthermore, a meta-analysis indicated that probiotics have the potential to reduce various indicators of kidney disease in individuals with diabetic nephropathy, including blood urea nitrogen, serum creatinine, cystatin C, and UACR [18]. According to a recent large survey, there was an inverse correlation between the intake of probiotics, prebiotics, or yogurt and the risk of CKD [8]. Previous studies have found that synbiotics can reduce uremic toxins such as cresyl sulfate, and improve fecal microbiota by reducing Ruminococcaceae and increasing Bifidobacteria in predialysis CKD patients [19]. Additionally, fresh fruits and vegetables are crucial sources of probiotics [10]. Of note, participants with CKD or those undergoing dialysis who consume fewer fruits and vegetables face an elevated risk of all-cause mortality [20, 21]. These studies provide support for exploring the prognostic impact of dietary live microbes on CKD patients. Our study is the first to show that individuals with CKD who consume high amounts of dietary live microbes have a reduced risk of all-cause and CVD mortality. While research on the advantageous effects of probiotics often focuses on examining specific strains, substantial evidence indicates that consuming adequate amounts of probiotics may yield health benefits irrespective of the strain [22]. Our results suggest that consuming moderate or high levels of dietary live microbes above approximately 110 g per day may have a positive impact on the prognosis of individuals with CKD. In light of these results, we propose the implementation of a long-term dietary plan for CKD patients, which poses no harm and aims to enhance their prognosis.

The gut-kidney axis [7, 23] offers explanatory mechanisms for the observed correlation. Microbes present in food enter the digestive system and establish themselves without being digested, thus modifying the composition of the gut microbiome [11, 12, 24]. A randomized clinical trial revealed that a high-fiber diet increases Lactobacillus and Bifidobacterium while reducing the number of opportunistic pathogens such as Desulfovibrio and Klebsiella in patients with type 2 diabetes [25]. Additionally, an interventional study indicated that a diet rich in fruits and vegetables can increase Prevotella, Lactobacillus, and Bifidobacterium [26]. Furthermore, a randomized cross-over study involving 14 healthy participants demonstrated that the abundance of Bifidobacterium species increased after consuming fermented dairy products [27]. CKD patients often have an impaired gut mucosal barrier function and disrupted gut microbiota, leading to elevated levels of aerobic bacteria and decreased levels of anaerobic bacteria [28]. Anaerobic bacteria, including Lactobacillus and Bifidobacterium, lead to carbohydrate fermentation to produce short-chain fatty acids, which effectively reduce pH and impede the growth of pathogenic bacteria [29, 30]. As a result, the levels of uremic toxins are diminished, alongside with the inhibition of inflammatory responses and oxidative stress, ultimately leading to a slower progression of renal function impairment [9, 30].

The present study has a substantial number of participants and strong statistical methods, nevertheless, it is crucial to acknowledge limitations in our study. Firstly, the classification of dietary live microbes was obtained by experts and through literature analysis. Secondly, a 24-h dietary recall might introduce recall biases, which would limit the accuracy of the findings. Thirdly, this analysis only evaluates the consumption of dietary live microbes once, without considering potential changes in diet during follow-up. Regrettably, the limitations of NHANES precluded dynamic adjustments. Fourthly, due to the limitations inherent in the NHANES database, our investigation examined only the association between dietary live microbe intake and mortality. It is important to further explore the relationship between dietary live microbe intake and other outcomes in patients with CKD, including renal failure. Finally, our main focus is on intake of food rich in live microbes rather than on the exact microbial counts. Despite accounting for confounding variables like HEI-2015, vitamin A, vitamin C, vitamin E, and carotenoids, additional elements, apart from live microbes, might also contribute to reduce mortality.

## Conclusion

Individuals who consume high levels of live microbes have a reduced risk of all-cause and CVD mortality.

## Supplementary Information

Below is the link to the electronic supplementary material.

Supplementary file1 (DOC 96 KB)

Supplementary file2 (DOC 56 KB)

## Data Availability

All data are publicly available and can be accessed at the NHANES (https://www.cdc.gov/nchs/nhanes/index.htm).
