# Supplementary material for: Relationship between dietary consumption of live microbes with mortality in adults with chronic kidney disease
Source: J Nephrol. 2025 Feb 12;38(6):1619–27. doi: 10.1007/s40620-025-02212-w (PMC12378498; doi:10.1007/s40620-025-02212-w)
Supplement: Supplementary file 1 — Supplementary file1 (DOC 96 KB) [file 40620_2025_2212_MOESM1_ESM.doc]

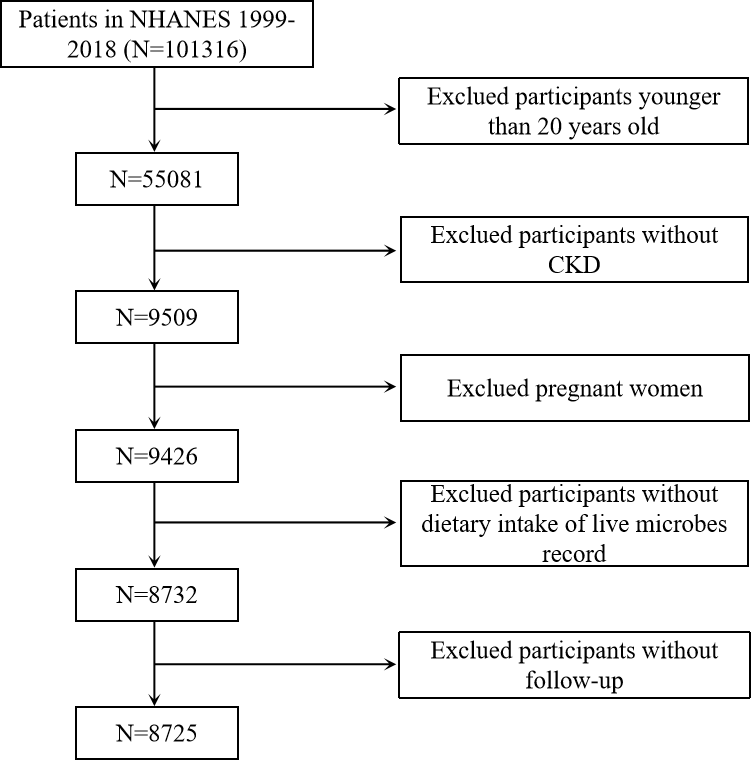


Sub figure 1. Flowchart of the sample selection from NHANES 1999–2018.

NHANES, National Health and Nutrition Examination Surveys; CKD, chronic kidney disease.
