# Supplementary material for: Relationship between dietary consumption of live microbes with mortality in adults with chronic kidney disease
Source: J Nephrol. 2025 Feb 12;38(6):1619–27. doi: 10.1007/s40620-025-02212-w (PMC12378498; doi:10.1007/s40620-025-02212-w)
Supplement: Supplementary file 2 — Supplementary file2 (DOC 56 KB) [file 40620_2025_2212_MOESM2_ESM.doc]

Supplementary Table 1 Sensitivity analysis of the association between Medhi dietary live microbes intake (per 100g) and the risk of mortality.

|  | Medhi | | | *P* for trend | Per one-unit increment in Medhi |
| --- | --- | --- | --- | --- | --- |
|  | Tertile 1 | Tertile 2 | Tertile 3 |
|  | HR (95% CI) | HR (95% CI) | HR (95% CI) |
| All-cause mortality |  |  |  |  |  |
| Model III+Prebiotic/Probiotic supplements | Reference | 0.82 (0.70, 0.95)^*^ | 0.74 (0.63, 0.87)^#^ | <0.001 | 0.91 (0.86, 0.97)^†^ |
| Model III+dietary factors^$^ | Reference | 0.88 (0.75, 1.02) | 0.83 (0.73, 0.95)^†^ | 0.005 | 0.94 (0.90, 0.98)^*^ |
| Model III with restrictions | | | | | |
| Excluding events within first 2 years | Reference | 0.90 (0.77, 1.05) | 0.79 (0.68, 0.91)^†^ | 0.001 | 0.92 (0.87, 0.96)^†^ |
| Censoring at 15 years follow-up | Reference | 0.86 (0.75, 0.99)^*^ | 0.79 (0.70, 0.90)^#^ | <0.001 | 0.92 (0.88, 0.96)^†^ |
| Excluding extreme energy intake% | Reference | 0.90 (0.78, 1.03) | 0.80 (0.71, 0.91)^#^ | <0.001 | 0.92 (0.87, 0.96)^#^ |
| CVD mortality |  |  |  |  |  |
| Model III+Prebiotic/Probiotic supplements | Reference | 0.82 (0.63, 1.06) | 0.65 (0.50, 0.86)^†^ | 0.002 | 0.89 (0.81, 0.98)^*^ |
| Model III+dietary factors^$^ | Reference | 0.88 (0.68, 1.14) | 0.76 (0.59, 0.97)^*^ | 0.025 | 0.92 (0.85, 0.99)^*^ |
| Model III with restrictions | | | | | |
| Excluding events within first 2 years | Reference | 0.92 (0.70, 1.20) | 0.78 (0.61, 1.00) | 0.051 | 0.91 (0.84, 0.98)^*^ |
| Censoring at 15 years follow-up | Reference | 0.88 (0.69, 1.14) | 0.75 (0.60, 0.93)^*^ | 0.009 | 0.91 (0.85, 0.97)^†^ |
| Excluding extreme energy intake% | Reference | 0.92 (0.71, 1.17) | 0.75 (0.60, 0.93)^*^ | 0.009 | 0.89 (0.83, 0.96)^†^ |

Medhi: >10^4^ CFU/g foods; HR, hazard ratios; 95% CI, 95% confidence interval; HEI-2015: healthy eating index-2015; CVD, cardiovascular diseases.

^$^dietary factors include the intakes of vitamin A, vitamin C, vitamin E, and carotenoids.

%We excluded participants with extreme energy intake levels below 500 kcal or exceeding 6000 kcal from the analysis.

Tertile 1 referred to individuals who consumed no live microbe food classified as Medhi. Tertile 2 included individuals who consumed Medhi in the 0-110 grams/d. Tertile 3 consisted of individuals with consumption exceeding 110 grams/d.

^*^P<0.05, ^†^P<0.01, ^#^P<0.001.
